# Breast percent density changes in digital mammography pre‐ and post‐radiotherapy

**DOI:** 10.1002/jmrs.788

**Published:** 2024-04-03

**Authors:** Sana Mohammadi, Sadegh Ghaderi, Mahdi Mohammadi, Hamid Ghaznavi, Kamal Mohammadian

**Affiliations:** ^1^ Department of Medicine Hamadan University of Medical Sciences Hamadan Iran; ^2^ Department of Neuroscience and Addiction Studies, School of Advanced Technologies in Medicine Tehran University of Medical Sciences Tehran Iran; ^3^ Department of Medical Physics and Biomedical Engineering, School of Medicine Tehran University of Medical Sciences Tehran Iran; ^4^ Department of Radiology, Faculty of Paramedical Sciences Kurdistan University of Medical Sciences Sanandaj Iran; ^5^ Department of Radiation Oncology Hamadan University of Medical Sciences, Mahdieh Center Hamadan Iran

**Keywords:** Breast cancer, breast density, image processing, mammography, percent density, radiotherapy

## Abstract

**Introduction:**

Breast cancer (BC), the most frequently diagnosed malignancy among women worldwide, presents a public health challenge and affects mortality rates. Breast‐conserving therapy (BCT) is a common treatment, but the risk from residual disease necessitates radiotherapy. Digital mammography monitors treatment response by identifying post‐operative and radiotherapy tissue alterations, but accurate assessment of mammographic density remains a challenge. This study used OpenBreast to measure percent density (PD), offering insights into changes in mammographic density before and after BCT with radiation therapy.

**Methods:**

This retrospective analysis included 92 female patients with BC who underwent BCT, chemotherapy, and radiotherapy, excluding those who received hormonal therapy or bilateral BCT. Percent/percentage density measurements were extracted using OpenBreast, an automated software that applies computational techniques to density analyses. Data were analysed at baseline, 3 months, and 15 months post‐treatment using standardised mean difference (SMD) with Cohen's *d*, chi‐square, and paired sample *t*‐tests. The predictive power of PD changes for BC was measured based on the receiver operating characteristic (ROC) curve analysis.

**Results:**

The mean age was 53.2 years. There were no significant differences in PD between the periods. Standardised mean difference analysis revealed no significant changes in the SMD for PD before treatment compared with 3‐ and 15‐months post‐treatment. Although PD increased numerically after radiotherapy, ROC analysis revealed optimal sensitivity at 15 months post‐treatment for detecting changes in breast density.

**Conclusions:**

This study utilised an automated breast density segmentation tool to assess the changes in mammographic density before and after BC treatment. No significant differences in the density were observed during the short‐term follow‐up period. However, the results suggest that quantitative density assessment could be valuable for long‐term monitoring of treatment effects. The study underscores the necessity for larger and longitudinal studies to accurately measure and validate the effectiveness of quantitative methods in clinical BC management.

## Graphical abstract

This study used OpenBreast software to analyse digital mammograms and quantitatively evaluate changes in breast percent density (PD) in women with breast cancer before and after radiotherapy, and at 3 and 15 months post‐treatment. These findings suggest that breast density continues to evolve over the first year following radiotherapy, highlighting the need for further research.

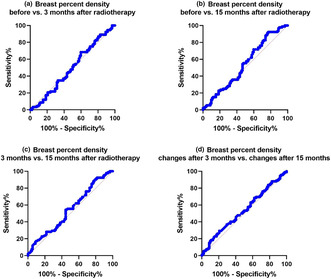

## Introduction

Cancer is a primary cause of mortality and a major impediment to increased life expectancy in every nation around the world.^1^ Breast cancer (BC) is a critical public health issue and currently the most frequently diagnosed malignancy in women worldwide.^1^, ^2^ With an expected 2.3 million new cases and 685,000 deaths in 2020, breast cancer (BC) has surpassed lung cancer as the most frequently diagnosed malignancy, followed by lung, colorectal, prostate, and stomach cancers.^3^ In 2020, BC accounted for 24.5% of all cancer cases and 15.5% of cancer‐related deaths among women, making it the most common type of cancer and leading cause of cancer‐related deaths in most countries.^4^ Breast‐conserving therapy (BCT) is a standard treatment for BC. Breast‐conserving therapy aims to excise cancerous tissue while sparing as much of the breast as possible; however, the inability to remove breast cancer in its entirety necessitates adjunctive treatments such as radiotherapy and chemotherapy to manage residual disease risk.^5^

Digital mammography, which uses low‐dose X‐rays to carefully examine the human breast, is widely recognised as the most reliable diagnostic tool for breast cancer diagnosis. This technology is excellent for detecting early‐stage BC, providing important insights into small changes in the breast tissue. These changes can include increased breast tissue density, changes in skin or structural features, and signs of fluid accumulation or scar formation, which can provide information about treatment response and aid in detecting recurrent cancers after breast‐conserving therapy (BCT).^6^ Moreover, post‐radiation therapy, density changes can be attributed to various factors including fibrosis, parenchymal oedema, postoperative collections, scar tissue formation, and skin thickening. These changes do not solely indicate increased fibroglandular tissue but also represent a complex interplay of treatment‐induced alterations in breast tissue composition.^7^, ^8^

Importantly, mammography can detect these changes and capture breast tissue density information. Advanced computational analysis of mammography images enables the quantification of breast tissue density, which is a powerful‐independent risk factor for BC.^9^ Percent density (PD) was measured by assessing the proportion of dense fibroglandular tissue in the breast.^10^, ^11^, ^12^ Until recently, digital mammography faced challenges in providing quantitative tissue density data, which is an important aspect of assessing BC risk.^13^, ^14^ However, recent advancements have begun to overcome these barriers, improving the analysis of density and allowing for the detection of subtle changes in post‐treatment tissue, as well as enhancing the detection of masses and overall assessment of recurrence risk.^13^, ^14^ This quantification is crucial, as studies have shown that women with higher PD values (>75%) have a significantly higher risk of developing BC (up to four–six times higher) than women with lower PD values (<5%).^12^, ^15^

Computational techniques, such as cumulus‐like interactive thresholding, have been introduced, offering a consistent means of measuring mammographic density that is less influenced by reader variability. Among these innovations is OpenBreast, which is a clinically validated software crafted for automated density measurements using an advanced thresholding technique. OpenBreast stages include data extraction from parenchymal analysis,^11^ digital mammography image standardisation,^10^ breast segmentation and identification of the chest wall,^16^ identifying the breast regions of interest (ROIs),^17^, ^18^ and breast density segmentation.^19^ Experts in medical imaging are increasingly interested in creating completely automated approaches that can reliably and quantitatively evaluate the PD levels. Estimation of PD may now be performed automatically using tools such as OpenBreast. OpenBreast is an example of a software tool that can estimate the PD values in a completely automated way.^12^

This study aims to analyse digital mammography images using OpenBreast to quantitatively evaluate changes in breast PD in women with BC before and after pre‐radiotherapy treatment, and at 3 months and 15 months post‐treatment.

## Methods

### Patient selection

This retrospective study included 92 female BC patients with a history of BCT surgery, chemotherapy (using six–eight rounds of anthracycline), and radiotherapy (2–3 weeks after chemotherapy). Radiotherapy was administered 2–3 weeks following the final cycle of chemotherapy to allow sufficient time for recovery from chemotherapy effects while capitalising on the increased sensitivity of residual tumour cells to radiation during the proliferative phase immediately after chemotherapy. PD was measured in the breast during BCT, and women who received hormonal therapy and bilateral BCT were excluded. This work was adapted from the MD thesis and supported by Hamadan University of Medical Sciences, Hamadan, Iran (Research Code: 30539). Ethical Code (approval ID): IR.UMSHA.REC.1398.434.

### Treatment design

The treatment procedure was designed using the 3D‐CRT technique, with a dose of 60 Gy in 30 sessions. The linear accelerator used was the Elekta Synergy Platform (Stockholm, Sweden) with an ISOgray treatment planning system. For patients with thin breast tissue, a six MV photon beam was used, whereas both six and 18 MV photon beams were applied to patients with thick breast tissue.

### OpenBreast

OpenBreast density analysis software is a parenchymal analysis framework that applies automated segmentation and feature extraction to mammographic images to quantify the breast density.^10^ Specifically, OpenBreast utilises a Cumulus‐like interactive thresholding technique to separate dense and non‐dense tissues in the breast region.^10^ By manually adjusting the intensity threshold in the mammogram, this method enables a human reader to separate dense parenchymal tissue from non‐dense tissue.^10^ Parenchymal analysis workflow, as described by OpenBreast, was applied.

#### Segmentation and ROI detection

Breast segmentation is a crucial step in quantifying breast tissue density using the OpenBreast density analysis software. This involves the identification of the breast area in a mammogram image. This process includes various tasks such as background detection, chest wall detection, and nipple recognition. OpenBreast developed at the Universidad Industrial de Santander's School of Electrical, Electronics, and Telecommunications Engineering in Boucaramanga, Colombia, provides a comprehensive framework for breast segmentation and ROI detection (www.github.com/spertuz/openbreast).^10^, ^11^, ^20^

#### Density analysis

Several medical and clinical studies have used interactive thresholding approach.^21^, ^22^, ^23^, ^24^, ^25^, ^26^, ^27^, ^28^, ^29^, ^30^ This strategy, an easy observer‐assisted approach called interactive thresholding, is a less time‐consuming alternative to planimetry for quantitatively assessing mammographically dense tissues. To separate the breast from the background, the observers first chose a grey value (*i*_EDGE_) as the cut‐off. To determine the pixels inside the breast projection, an edge detection algorithm examined the images in a grid pattern extending from the chest wall to the nipple. One way to determine the size of each area is to examine the histogram of grey‐level frequencies from the pixels within the breast. The histogram of grey‐level frequencies displays the distribution of the grey‐level values within the breast region. Each grey‐level value corresponds to a frequency value that indicates the number of pixels within the breast region that contain a specific grey‐level value. By examining the histogram, we can easily calculate the number of pixels with a particular grey‐level value, which is necessary for determining the projected area of the breast, using equation 1. In equation 1, ‘hi’ represents the number of pixels with grey‐level value ‘*i*’ in the histogram, and ‘*i*_EDGE_’ and ‘*i*_MAX_’ represent the lower and upper grey‐level values, respectively, used to threshold the breast region. The area under the histogram between the grey‐level values ‘*i*_EDGE_’ and ‘*i*_MAX_’ represents the sum of all pixels within the breast region with grey‐level values within this range, which is used to determine the projected area of the breast.

(1) $$\text{Projected Area}=\sum_{i=i_{\text{EDGE}}}^{i_{\mathrm{MAX}}}\mathrm{hi}$$

A second threshold value, *i*_DY_, was selected to identify areas with a high mammographic density. This threshold value was used to distinguish dense from non‐dense tissues in the mammogram. Pixels with grayscale values greater than or equal to *i*_DY_ represent dense tissues. In addition, the pectoral muscle was removed from the final result using a masking tool provided in the OpenBreast toolbox. In mammography, the density of breast tissue is estimated based on the grayscale values of pixels in the mammogram. Pixels with grayscale values greater than or equal to *i*_DY_ were considered to represent dense tissue, and the region below the histogram that was larger than this threshold was used for density estimates. The mammographic PD value was calculated by multiplying the fraction of dense tissue (calculated as the number of pixels with grayscale values greater than or equal to i_DY_ divided by the total number of breast pixels) by total breast area. This is expressed in (2).

(2) $$\mathrm{PD}=\frac{\sum_{i=i_{\mathrm{DY}}}^{i_{\mathrm{MAX}}}\mathrm{hi}}{\text{Projected Area}}\times 100\%$$

### Analysis of mammographic images

**Figure 1:**
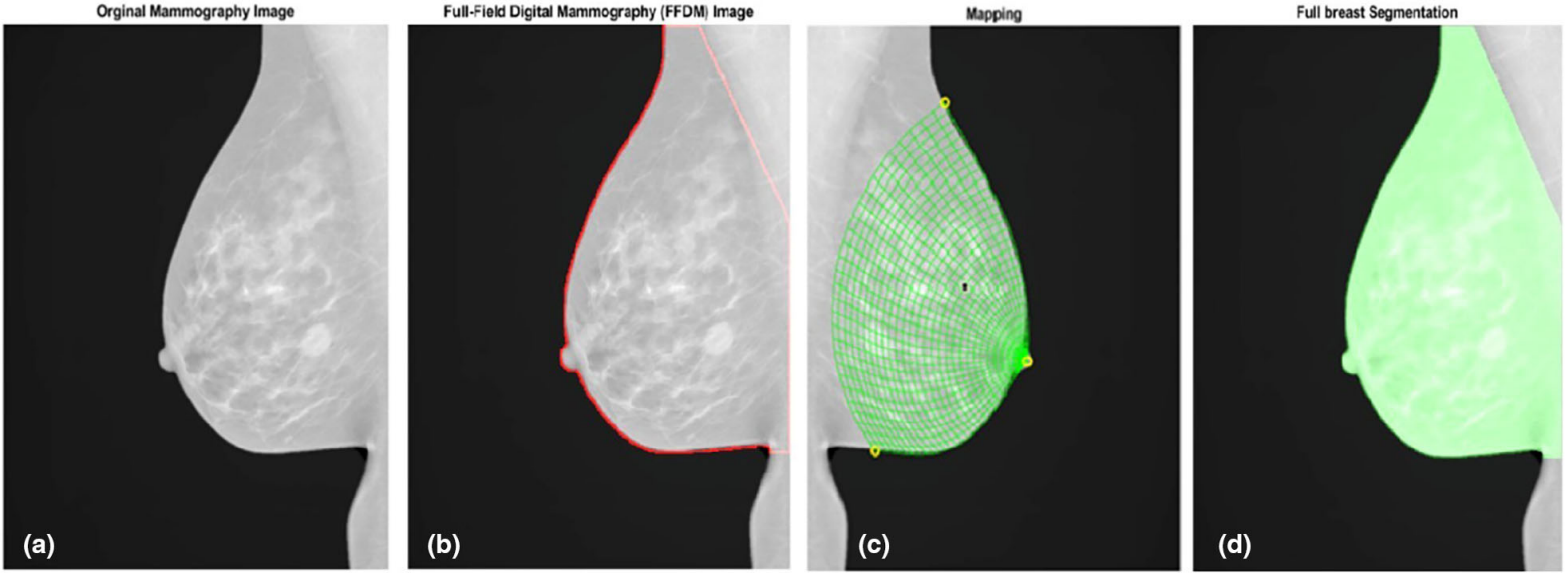
Percent density (PD) measurements process by Openbreast in MATLAB.

**Figure 2:**
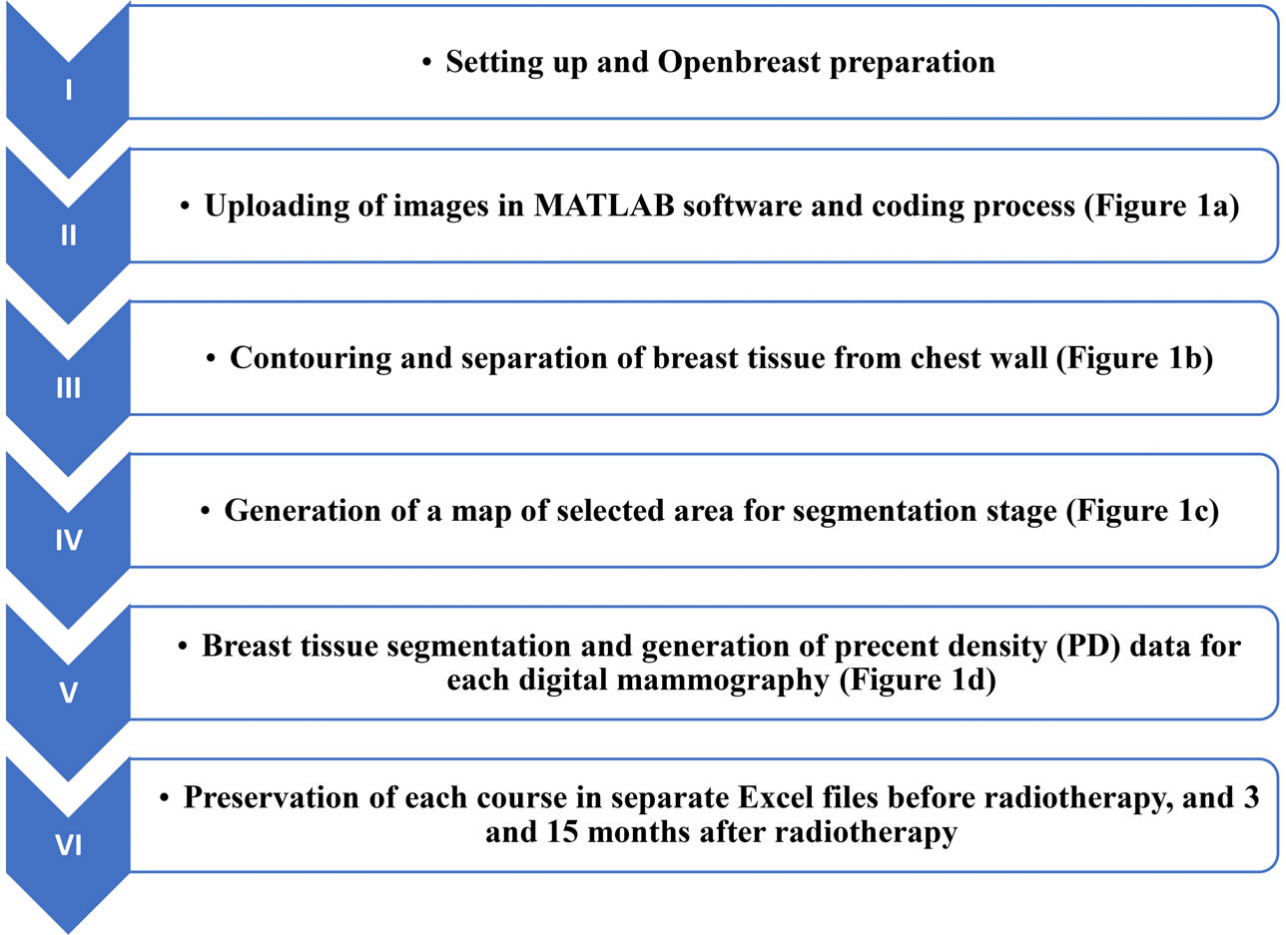
Analysis process flow chart of mammographic images analysis.

Mammography was conducted before, as well as at the 3 and 15‐month time points, following breast radiotherapy. To assess alterations in breast tissue before and after radiotherapy, MATLAB 2021a software was used for the analysis. The Openbreast toolbox was utilised for the analysis, with the coding implemented in the MATLAB software. Breast tissue density was assessed at both pre‐ and post‐treatment stages (Fig. 1). The steps involved in this process are illustrated in the flowchart in (Fig. 2).

### Statistical analysis

Collected data were analysed using SPSS™ software (v. 20) using descriptive statistics, Chi‐square, and Paired Sample *T*‐test samples. Statistical significance was set at *P* < 0.05, and the data are expressed as the mean ± SD. Stata version 17 (StataCorp, College Station, TX, USA) was also used for the statistical analysis. The standardised mean difference (SMD) was used to analyse the mean PD among patients before treatment, 3 months after radiotherapy, and 15 months after radiotherapy. Cohen's *d* cut‐off values (0.2, 0.5, and 0.8) were applied.^31^

## Results

**Figure 3:**
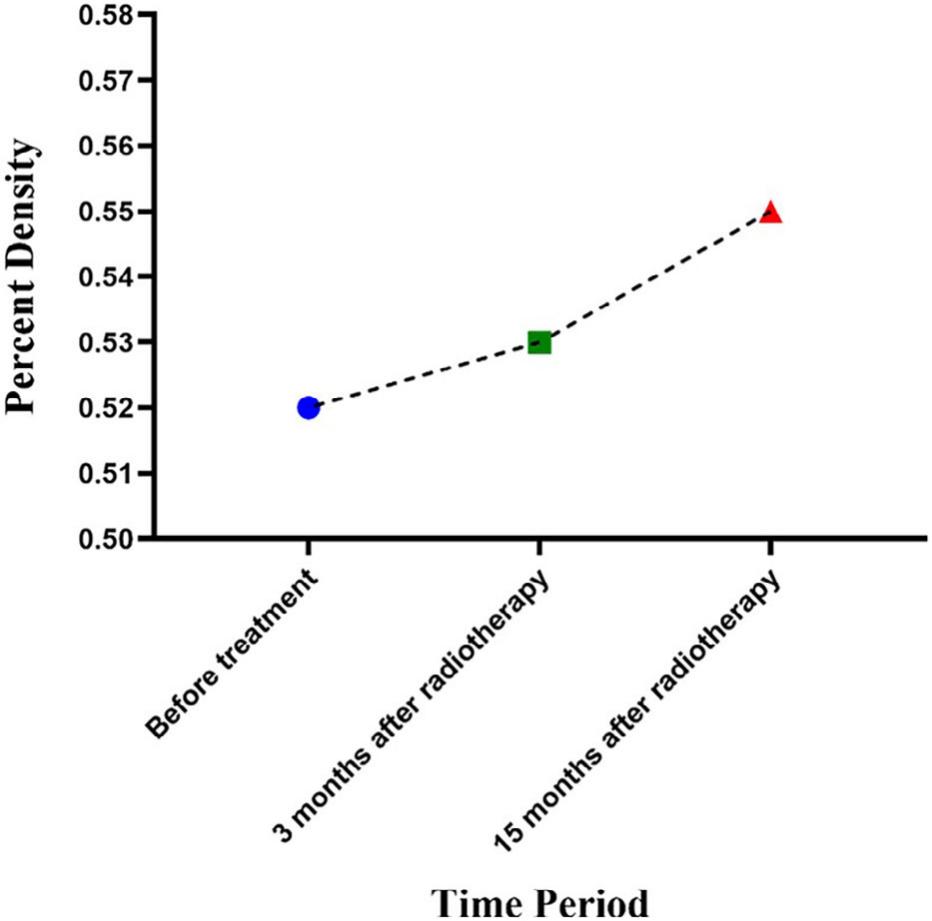
Linear chart of changes in breast density in three time periods.

The mean age of the patients was 53.2 ± 7.6 years. Tables 1 and 2 present the mean breast densities before and after RT, as well as the changes in breast PD in different time periods, respectively. Changes in breast density are shown in Figure 3.

**Table 1:** Determination of breast tissue density in patients with breast cancer

Time period	Percent density (PD) (Mean ± SD)	Mean difference	*P*‐value (Before vs. After Radiotherapy)
Before radiotherapy	0.52 ± 0.13	−0.002	1
3 months after radiotherapy	0.53 ± 0.09	−0.023	0.47
15 months after radiotherapy	0.55 ± 0.09	−0.02	0.17

**Table 2:** Breast percent density changes in different time periods

	Decreasing cases n (%)	Mean reduction (%)	Increasing cases n (%)	Mean increase (%)	No change cases n (%)
Before versus 3 months after	49 (53.2%)	8.68	41 (44.5%)	10.91	2 (2.3%)
Before versus 15 months after	46 (52%)	8.54	44 (47.7%)	13.63	2 (2.3%)

**Figure 4:**
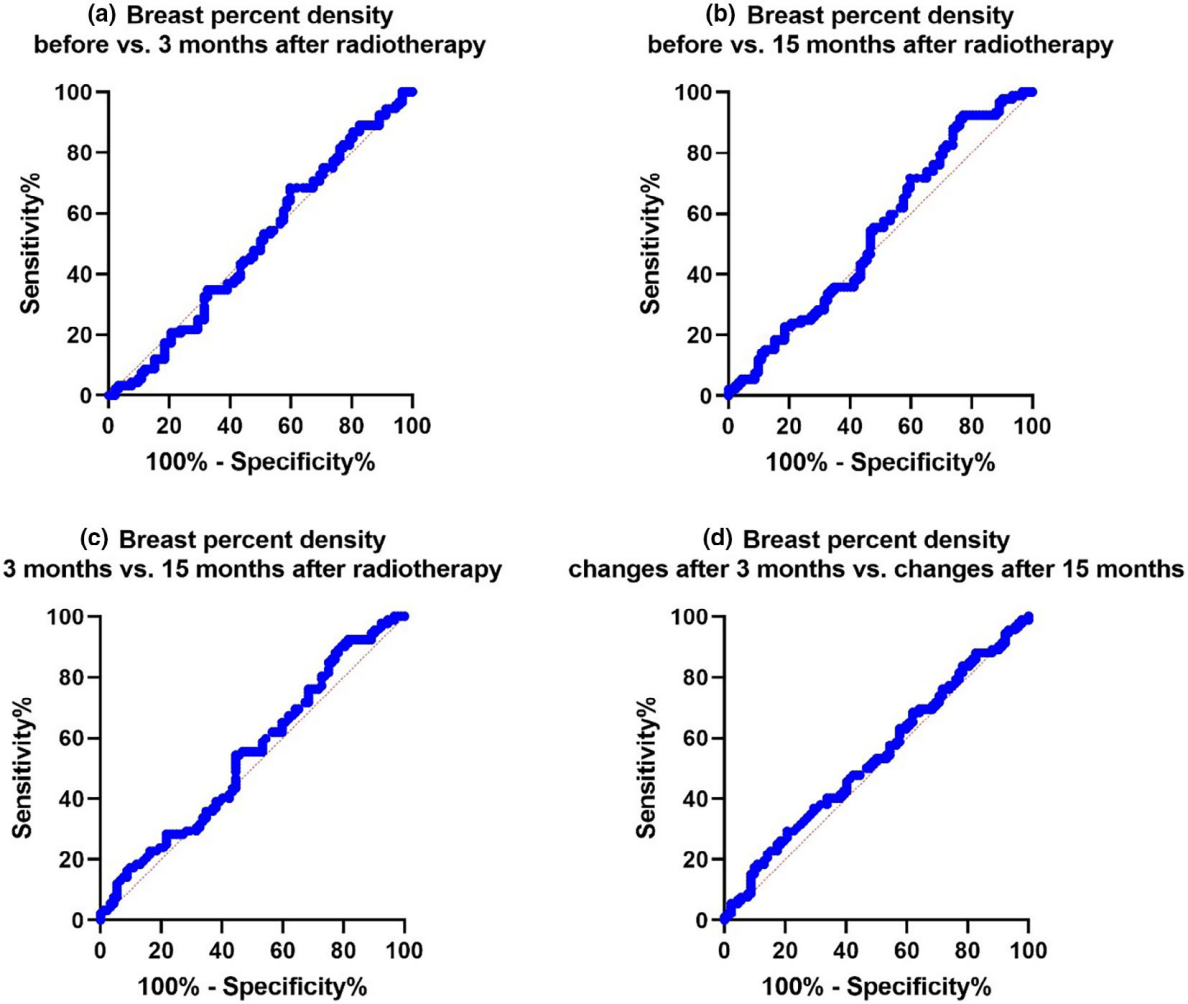
ROC curves of breast percent density in different time periods.

We conducted a comparative receiver operating characteristic (ROC) analysis to compare changes in breast density across the four different states, as illustrated in Figure 4. These comparisons included density changes ‘before versus 3 months after’, ‘before versus 15 months after’, ‘3 months after versus 15 months after’, and comparisons of ‘PD changes after 3 months versus PD changes after 15 months’.

These results are shown in Figure 4a–d, respectively. The sensitivity for ‘before versus 3 months after’ radiotherapy is 68.48%, while for ‘before versus 15 months after’ radiotherapy, it was 92.39%. Maximum sensitivity was achieved ‘before versus 15 months after’. The maximum specificity was seen in ‘before versus 3 months after’, and the specificities for ‘before versus 3 months after’ and ‘before versus 15 months after’ and ‘3 months after versus 15 months after’, but the specificity changed. Therefore, it can be assumed that the sensitivity and specificity values are not high. The sensitivity and specificity of PD changes after 3 months versus PD changes after 15 months were 29.35% and 79.35%, respectively. Again, the sensitivity and specificity levels were not high at the same time. If the objective was to compare PD before and after therapy, longer time intervals for follow‐up and screening for PD changes would be more appropriate. Our results also showed that the higher sensitivity value (92.39%) at 15 months compared with at 3 months of follow‐up is likely linked to PD changes in breast tissue density (Table 3).

**Table 3:** The receiver operating characteristic (ROC) analysis of breast percent density in different time periods

Parameter	AUC	Cut‐off values	Sensitivity (%)	Specificity (%)
Before versus 3 months after	0.5002	0.5130	68.48	40.22
Before versus 15 months after	0.5401	0.4446	92.39	22.83
3 months after versus 15 months after	0.5434	0.4448	92.39	18.48
PD changes after 3 months versus PD changes after 15 months	0.5357	6.533	29.35	79.35

The study did not report any significant change in the SMD between PD before treatment versus PD 3 months after radiotherapy (−0.08, 95% CI −0.37 to 0.19), PD before treatment versus PD 15 months after radiotherapy (−0.26, 95% CI −0.55 to 0.22), and PD 3 months after radiotherapy versus PD 15 months after radiotherapy (−0.22, 95% CI −0.51 to 0.06).

## Discussion

This study evaluated the quantitative changes in breast density on digital mammography before and after radiotherapy for BC. The key finding was that there were no statistically significant differences in percent breast density at 3 months or 15 months after radiotherapy compared to baseline. However, there was a numerical increase in mean PD at both time points after radiotherapy. The lack of significant differences could be due to the relatively small sample size of 92 patients, resulting in insufficient power to detect small underlying changes in the density. Nevertheless, the numerical increase in breast density observed here aligns with prior evidence suggesting that radiotherapy can increase breast density, likely due to acute inflammatory effects and subsequent fibrous tissue formation.^32^, ^33^, ^34^, ^35^ Denser breast tissue on mammography makes the detection of small tumours more challenging, and is an independent risk factor for BC.^36^ Although mammographic density is a reliable indicator of BC risk in populations, one study suggested that it cannot accurately predict BC risk in individuals. Factors such as age, parity, menopausal status, race/ethnicity, and body mass index (BMI) can influence mammographic density.^32^, ^33^, ^34^

ROC analysis indicated that a longer interval of 15 months after radiotherapy provided higher sensitivity for detecting changes in density than just 3 months. This suggests that breast density continues to evolve over the first year after radiotherapy. However, even after 15 months, specificity remained low. Larger prospective studies with radiotherapy and non‐radiotherapy control groups are required to define the optimal timeline and threshold for clinically meaningful density changes.

According to a previous study, mammographic PD is a highly reliable quantitative value for breast density evaluation as it has a highly acceptable intra‐rater agreement.^37^ Similarly, another study found that OpenBreast showed significant levels of agreement with manual segmentation.^20^ These findings support the validity of our method for assessing the changes in breast tissue density over time. A study found that mammographic density was significantly associated with survival rates in BC patients with.^38^ However, our study did not find a clear association between PD and radiotherapy after 15 months, suggesting that further research is needed to investigate this relationship more thoroughly. Similar to our study, another study found an increase in parenchymal density and skin thickness of the breast region after radiotherapy, similar to our study.^39^

A primary study observed epithelial and vascular alterations in subjects treated with radiotherapy as well as soft tissue changes in both the treatment and control groups.^40^ Another study reported that decreased mammographic density after BC diagnosis appears to be a prognostic marker for improving the long‐term survival rate during adjuvant Tamoxifen treatment.^41^ However, our study found an overall increase in breast tissue density after radiotherapy, which may be due to differences in treatment protocols or patient populations.

A previous study showed that high breast density and obesity are essential independent predictors of late local recurrence after BCT and radiotherapy.^42^ However, another group found conflicting data on the relationship between high mammographic density and various cancer‐related factors, underlining the need for further studies.^43^

A recent study used Volpara™ software to analyse mammographic density in Japanese patients and found that measuring mammographic density could enhance the accuracy of BC detection.^44^ Our study also employed breast densitometry software to quantify density, in line with the growing recognition of the importance of standardised, automated methods for assessing breast tissue density. Another study demonstrated that the automated percentage of breast density measurement is a viable option for future testing in a clinical setting,^45^ which is in line with our research methodology. Further, there is a need for standardised methods to limit the impact of individual readers' subjective assessments of breast density.^46^ Using the OpenBreast tool, we aimed to minimise this impact and provide a more objective assessment of breast tissue density changes over time.

Our findings underscore the importance of using standardised automated methods to assess breast tissue density in clinical practice and research. Our study had several strengths, including the use of digital mammography to quantify breast tissue density; the inclusion of patients undergoing surgery, chemotherapy, and radiotherapy; and the evaluation of PD changes over time. However, this study had some limitations. This study had a relatively small sample size, which may have limited the statistical power of the analysis. Although we observed a slight increase in PD at 15 months after radiotherapy, longer follow‐up periods may be necessary to detect any significant changes in PD. In addition, this study did not consider other factors that may influence breast tissue density, such as age, menopausal status, or hormonal therapy. Future studies with larger sample sizes, longer follow‐up periods, and more comprehensive assessments of breast tissue density and other relevant clinical factors are necessary to fully understand the relationship between breast tissue density and radiotherapy outcomes.

## Conclusions

This study investigated changes in breast tissue density using digital mammography in women after BC treatment. We found no statistically significant differences in PD at either 3 or 15‐months post‐radiotherapy compared to baseline. The minor numerical increase in PD did not translate into statistical significance, possibly due to the small sample size and the retrospective nature of the study. Moreover, this increase aligns with the known radiotherapy effect of density increase through inflammation and fibrosis, which can complicate tumour detection in mammography. This study suggests that PD changes may continue over the first year after radiotherapy. Our study underscores the need for larger studies with longer follow‐up periods to enhance our understanding of the changes in PD. It also highlights the potential benefits of standardised quantitative breast density assessments for monitoring the long‐term effects of radiotherapy.

## Author's Contributions

S.G., S.M., and K.M. contributed to the conception and design of the study. S.M., S.G., and K.M. contributed to the data collection. S.G., S.M., M.M., and H.G. drafted the text and prepared a graphical table of the content. S.M., S.G., and K.M. contributed to revising the manuscript.

## Conflict of Interest

The authors declare no financial or other conflicts of interest.

## Ethical Approval

This work was adapted from the MD thesis and supported by Hamadan University of Medical Sciences, Hamadan, Iran (Research Code: 30539). Ethical Code (approval ID): IR.UMSHA.REC.1398.434.

## Data Availability

The data that support the findings of this study are available from the corresponding author upon reasonable request.

## References

[1] Mohammadi M , Mohammadi S , Hadizadeh H , et al. Brain metastases from breast cancer using magnetic resonance imaging: A systematic review. J Med Radiat Sci 2023; 71: 133–141. 37563948 10.1002/jmrs.715 PMC10920938

[2] Asbeutah AM , AlMajran AA , Brindhaban A , Asbeutah SA . Comparison of radiation doses between diagnostic full‐field digital mammography (FFDM) and digital breast tomosynthesis (DBT): A clinical study. J Med Radiat Sci 2020; 67: 185–192. 32495513 10.1002/jmrs.405 PMC7476200

[3] Arnold M , Morgan E , Rumgay H , et al. Current and future burden of breast cancer: Global statistics for 2020 and 2040. Breast Off J Eur Soc Mastology 2022; 66: 15–23. 10.1016/j.breast.2022.08.010 PMC9465273 36084384

[4] Lei S , Zheng R , Zhang S , et al. Global patterns of breast cancer incidence and mortality: A population‐based cancer registry data analysis from 2000 to 2020. Cancer Commun 2021; 41: 1183–1194. 10.1002/cac2.12207 PMC8626596 34399040

[5] Pfob A , Dubsky P . The underused potential of breast conserving therapy after neoadjuvant system treatment – Causes and solutions. Breast 2023; 67: 110–115. 36669994 10.1016/j.breast.2023.01.008 PMC9982288

[6] Mall S , Lewis S , Brennan P , Noakes J , Mello‐Thoms C . The role of digital breast tomosynthesis in the breast assessment clinic: A review. J Med Radiat Sci 2017; 64: 203–211. 28374502 10.1002/jmrs.230 PMC5587657

[7] Verbelen H , Tjalma W , Dombrecht D , Gebruers N . Breast edema, from diagnosis to treatment: State of the art. Arch Phys Ther 2021; 11: 8. 10.1186/s40945-021-00103-4 PMC8006345 33775252

[8] Yi A , Kim HH , Shin HJ , Huh MO , Ahn SD , Seo BK . Radiation‐induced complications after breast cancer radiation therapy: A pictorial review of multimodality imaging findings. Korean J Radiol 2009; 10: 496–507. 19721835 10.3348/kjr.2009.10.5.496 PMC2731868

[9] Galli V , Pini M , De Metrio D , de Bianchi PS , Bucchi L . An image quality review programme in a population‐based mammography screening service. J Med Radiat Sci 2021; 68: 253–259. 34085397 10.1002/jmrs.487 PMC8424329

[10] Pertuz S , Sassi A , Holli‐Helenius K , et al. Clinical evaluation of a fully‐automated parenchymal analysis software for breast cancer risk assessment: A pilot study in a Finnish sample. Eur J Radiol 2019; 121: 108710. 31689665 10.1016/j.ejrad.2019.108710

[11] Pertuz S , Torres GF , Tamimi R , Kamarainen J . Open framework for mammography‐based breast cancer risk assessment. 2019 IEEE EMBS Int Conf Biomed Health Inform BHI 2019 – Proc. 2019.

[12] Gudhe NR , Behravan H , Sudah M , et al. Area‐based breast percentage density estimation in mammograms using weight‐adaptive multitask learning. Sci Rep 2022; 12: 1–19. 35835933 10.1038/s41598-022-16141-2 PMC9283472

[13] Ahn JS , Shin S , Yang SA , et al. Artificial intelligence in breast cancer diagnosis and personalized medicine. J Breast Cancer 2023; 26: 405–435. 37926067 10.4048/jbc.2023.26.e45 PMC10625863

[14] Pinkert MA , Salkowski LR , Keely PJ , Hall TJ , Block WF , Eliceiri KW . Review of quantitative multiscale imaging of breast cancer. J Med Imaging 2018; 5: 010901. 10.1117/1.JMI.5.1.010901 PMC5777512 29392158

[15] Advani SM , Zhu W , Demb J , et al. Association of breast density with breast cancer risk among women aged 65 years or older by age group and body mass index. JAMA Netw Open 2021; 4: 2122810. 10.1001/jamanetworkopen.2021.22810 PMC8391100 34436608

[16] Keller BM , Nathan DL , Wang Y , et al. Estimation of breast percent density in raw and processed full field digital mammography images via adaptive fuzzy c‐means clustering and support vector machine segmentation. Med Phys 2012; 39: 4903–4917. 22894417 10.1118/1.4736530 PMC3416877

[17] Pertuz S , Julia C , Puig D . A novel mammography image representation framework with application to image registration. In 2014 22nd International Conference on Pattern Recognition. IEEE, Stockholm, Sweden, 2014; 3292–3297.

[18] Torres GF , Pertuz S . Automatic detection of the retroareolar region in X‐ray mammography images. In VII Latin American Congress on Biomedical Engineering CLAIB 2016. October 26th-28th, 2016. Springer, Bucaramanga, Colombia, 2017; 157–160.

[19] Torres GF , Sassi A , Arponen O , et al. Morphological area gradient: System‐independent dense tissue segmentation in mammography images. In 2019 41st Annual International Conference of the IEEE Engineering in Medicine and Biology Society (EMBC) 2019 July 23. IEEE, Berlin, Germany, 2019; 4855–4858. 10.1109/EMBC.2019.8857320 31946948

[20] Sansone M , Marrone S , Di Salvio G , et al. Comparison between two packages for pectoral muscle removal on mammographic images. Radiol Med (Torino) 2022; 127: 848–856. 35816260 10.1007/s11547-022-01521-5 PMC9349098

[21] Boyd NF , Martin LJ , Bronskill M , Yaffe MJ , Duric N , Minkin S . Breast tissue composition and susceptibility to breast cancer. J Natl Cancer Inst 2010; 102: 1224–1237. 20616353 10.1093/jnci/djq239 PMC2923218

[22] Chiarelli AM , Kirsh VA , Klar NS , et al. Influence of patterns of hormone replacement therapy use and mammographic density on breast cancer detection. Cancer Epidemiol Biomarkers Prev 2006; 15: 1856–1862. 17035392 10.1158/1055-9965.EPI-06-0290

[23] Palomares MR , Machia JRB , Lehman CD , Daling JR , McTiernan A . Mammographic density correlation with gail model breast cancer risk estimates and component risk factors. Cancer Epidemiol Biomarkers Prev 2006; 15: 1324–1330. 16835331 10.1158/1055-9965.EPI-05-0689

[24] Mitchell G , Antoniou AC , Warren R , et al. Mammographic density and breast cancer risk in BRCA1 and BRCA2 mutation carriers. Cancer Res 2006; 66: 1866–1872. 16452249 10.1158/0008-5472.CAN-05-3368

[25] Gram IT , Bremnes Y , Ursin G , Maskarinec G , Bjurstam N , Lund E . Percentage density, Wolfe's and Tabár's mammographic patterns: Agreement and association with risk factors for breast cancer. Breast Cancer Res 2005; 7: R854–R861. 16168132 10.1186/bcr1308 PMC1242160

[26] Buist DSM , Aiello EJ , Miglioretti DL , White E . Mammographic breast density, dense area, and breast area differences by phase in the menstrual cycle. Cancer Epidemiol Biomarkers Prev 2006; 15: 2303–2306. 17119062 10.1158/1055-9965.EPI-06-0475

[27] Llobet R , Pollán M , Antón J , et al. Semi‐automated and fully automated mammographic density measurement and breast cancer risk prediction. Comput Methods Prog Biomed 2014; 116: 105–115. 10.1016/j.cmpb.2014.01.021 24636804

[28] Juneja P , Harris EJ , Kirby AM , Evans PM . Adaptive breast radiation therapy using modeling of tissue mechanics: A breast tissue segmentation study. Int J Radiat Oncol Biol Phys 2012; 84: e419–e425. 22717244 10.1016/j.ijrobp.2012.05.014

[29] Vachon CM , Scott CG , Fasching PA , et al. Common breast cancer susceptibility variants in LSP1 and RAD51L1 are associated with mammographic density measures that predict breast cancer risk. Cancer Epidemiol Biomarkers Prev 2012; 21: 1156–1166. 22454379 10.1158/1055-9965.EPI-12-0066 PMC3569092

[30] Woolcott CG , Koga K , Conroy SM , et al. Mammographic density, parity and age at first birth, and risk of breast cancer: An analysis of four case‐control studies. Breast Cancer Res Treat 2012; 132: 1163–1171. 22222356 10.1007/s10549-011-1929-9 PMC3336030

[31] Cohen J . Statistical Power Analysis for the Behavioral Sciences, 2nd edn. Routledge, New York, 1988; 567.

[32] Tossas‐Milligan K , Shalabi S , Jones V , et al. Mammographic density: Intersection of advocacy, science, and clinical practice. Curr Breast Cancer Rep 2019; 11: 100–110. 33312342 10.1007/s12609-019-00316-4 PMC7728377

[33] Boyd NF , Martin LJ , Yaffe MJ , Minkin S . Mammographic density and breast cancer risk: Current understanding and future prospects. Breast Cancer Res 2011; 13: 223. 22114898 10.1186/bcr2942 PMC3326547

[34] Boyd NF , Guo H , Martin LJ , et al. Mammographic density and the risk and detection of breast cancer. N Engl J Med 2007; 356: 227–236. 17229950 10.1056/NEJMoa062790

[35] Kanbayti IH , Rae WID , McEntee MF , Ekpo EU . Mammographic density changes following BC treatment. Clin Imaging 2021; 76: 88–97. 33578136 10.1016/j.clinimag.2021.01.002

[36] Bodewes FTH , van Asselt AA , Dorrius MD , Greuter MJW , de Bock GH . Mammographic breast density and the risk of breast cancer: A systematic review and meta‐analysis. Breast Off J Eur Soc Mastology 2022; 66: 62–68. 10.1016/j.breast.2022.09.007 PMC9530665 36183671

[37] Sohn G , Lee JW , Park SW , et al. Reliability of the percent density in digital mammography with a semi‐automated thresholding method. J Breast Cancer 2014; 17: 174–179. 25013440 10.4048/jbc.2014.17.2.174 PMC4090321

[38] Maskarinec G , Pagano IS , Little MA , Conroy SM , Park SY , Kolonel LN . Mammographic density as a predictor of breast cancer survival: The Multiethnic Cohort. Breast Cancer Res 2013; 15: R7. 23339436 10.1186/bcr3378 PMC3672725

[39] Braw M , Erlandsson I , Ewers SB , Samuelsson L . Mammographic follow‐up after breast conserving surgery and postoperative radiotherapy without boost irradiation for mammary carcinoma. Acta Radiol Stockh Swed 1987 1991; 32: 398–402. 1910995

[40] Girling AC , Hanby AM , Millis RR . Radiation and other pathological changes in breast tissue after conservation treatment for carcinoma. J Clin Pathol 1990; 43: 152–156. 2318992 10.1136/jcp.43.2.152 PMC502299

[41] Li J , Humphreys K , Eriksson L , Edgren G , Czene K , Hall P . Mammographic density reduction is a prognostic marker of response to adjuvant tamoxifen therapy in postmenopausal patients with breast cancer. J Clin Oncol Off J Am Soc Clin Oncol 2013; 31: 2249–2256. 10.1200/JCO.2012.44.5015 PMC3677838 23610119

[42] Park CC , Rembert J , Chew K , Moore D , Kerlikowske K . High mammographic breast density is independent predictor of local but not distant recurrence after lumpectomy and radiotherapy for invasive breast cancer. Int J Radiat Oncol Biol Phys 2009; 73: 75–79. 18692323 10.1016/j.ijrobp.2008.04.007

[43] Shawky MS , Huo CW , Henderson MA , Redfern A , Britt K , Thompson EW . A review of the influence of mammographic density on breast cancer clinical and pathological phenotype. Breast Cancer Res Treat 2019; 177: 251–276. 31177342 10.1007/s10549-019-05300-1

[44] Yoshida R , Yamauchi T , Akashi‐Tanaka S , et al. Optimal breast density characterization using a three‐dimensional automated breast densitometry system. Curr Oncol Tor Ont 2021; 28: 5384–5394. 10.3390/curroncol28060448 PMC8700257 34940087

[45] Fowler EEE , Vachon CM , Scott CG , Sellers TA , Heine JJ . Automated percentage of breast density measurements for full‐field digital mammography applications. Acad Radiol 2014; 21: 958–970. 25018067 10.1016/j.acra.2014.04.006 PMC4166439

[46] Keller BM , Nathan DL , Gavenonis SC , Chen J , Conant EF , Kontos D . Reader variability in breast density estimation from full‐field digital mammograms: The effect of image postprocessing on relative and absolute measures. Acad Radiol 2013; 20: 560–568. 23465381 10.1016/j.acra.2013.01.003 PMC3673702

